# Contemporary trends in operative vaginal delivery and obstetric anal sphincter injuries from 2016 to 2023

**DOI:** 10.1002/pmf2.70063

**Published:** 2025-06-30

**Authors:** Minhazur R. Sarker, Elizabeth N. Teal, Rachel Wiley, Ukachi Emeruwa, Joyce Kim, George Arida, Marc Engelbert, Cynthia Gyamfi‐Bannerman, Timothy Wen

**Affiliations:** ^1^ Department of Obstetrics Gynecology and Reproductive Science University of California San Diego San Diego California USA; ^2^ Department of Obstetrics Gynecology and Reproductive Science Mount Sinai Health System & Icahn School of Medicine at Mount Sinai New York New York USA; ^3^ Department of Medicine University of California San Diego San Diego California USA

**Keywords:** FAVD, forceps, OASIS, operative vaginal delivery, vacuum, VAVD

## Abstract

**Introduction:**

Operative vaginal delivery (OVD) has experienced a decline, primarily driven by decreasing rates of forceps‐assisted vaginal delivery (FAVD). FAVD rates have been suspected to be declining to a point where recovery of this skill may be improbable. While there are numerous reasons for this decline, ranging from lack of training to patient preferences, the same period has been suspected to have worsening morbidity with FAVD. Concerns have been raised with respect to obstetric anal sphincter injuries (OASIS). This study aims to characterize national trends in OVD and risk factors for associated OASIS between 2016 and 2023 by assessing changes in OVD utilization and the associated risk of OASIS.

**Materials and Methods:**

This is a cross‐sectional study using National Vital Statistics System birth certificate data between 2016 and 2023 to identify temporal trends in OVD and OASIS rates. Liveborn deliveries undergoing trial of labor from 34 weeks and 0 days to 42 weeks and 6 days to patients aged 16–50 years were included. Pregnancies with multifetal gestations, fetal anomalies, non‐vertex presentations, and unknown mode of delivery were excluded. Temporal trends in OVD and OASIS were assessed using Joinpoint regression. Multivariable logistic regression models were fit to assess the association between OASIS and OVD, adjusting for maternal demographics and comorbidities.

**Results:**

Of the 21,191,398 liveborn deliveries identified, 18,054,141 (85.2%) were spontaneous vaginal births, 2,303,168 (10.9%) were cesarean after trial of labor, 136,927 (0.6%) were FAVDs, and 697,162 (3.3%) were vacuum‐assisted vaginal deliveries (VAVDs) with a OVD composite of 3.9%. OVD rates decreased significantly from 4.6% to 4.1% (annual average percent change [AAPC]: −1.6%, 95% CI: −1.9% to −1.4%). Specifically, FAVD rates declined from 0.8% to 0.6% (AAPC: −3.1%, 95% CI: −3.7% to −2.6%) and VAVD rates declined from 3.8% to 3.4% (AAPC: −1.4%, 95%CI: −1.7% to 1.1%). OASIS rates in the overall group did not significantly change (AAPC: 1.4%, 95%CI: −2.1% to 4.4%), although among FAVDs, there was a significant increase in rates of OASIS from 9.3% in 2016 to 14.0% in 2023 (AAPC: 4.8%, 95%CI: 2.3% to 7.0%). Adjusted multivariable logistic regression noted higher likelihood of OASIS in FAVD (aOR: 9.50, 95%CI: 9.33 to 9.66), and VAVD (aOR: 3.90, 95%CI: 3.85 to 3.94) when compared to spontaneous vaginal birth, adjusting for maternal age, maternal BMI, and maternal comorbidities including diabetes and hypertensive disorders.

**Conclusion:**

The primary finding in this study was a national decline in OVD with significantly greater declines in FAVD and an increasing rate of OASIS among FAVD. Whether targeted interventions to enhance training in safe FAVD may increase proficiency in this technique remains to be seen.

## 1 INTRODUCTION

Operative vaginal delivery (OVD) has experienced a contemporaneous decline, primarily driven by decreasing rates of forceps‐assisted vaginal delivery (FAVD) but also decreasing rates of vacuum‐assisted vaginal delivery (VAVD) [1, 2]. Specifically, FAVD rates dropped from 10% of all vaginal deliveries in the 1990s to 3% by 2014 [1, 2]. This decrease has mirrored a significant decline in the average number of FAVD reported by trainees in obstetrics and gynecology from the early 2000s to the mid‐2010s [1, 3, 4]. OVD rates are approaching a phase of critical slowing, also known as a tipping point, beyond which recovery of this skill may be improbable [5].

The decline is likely multifactorial and suspected to range from lack of training, fear of litigation, patient preferences, and worsening comorbidities among patients [1, 6]. In the United States, while OVD rates declined, cesarean deliveries among nulliparous, term, singleton, and vertex (NTSV) pregnancies have demonstrated an increase. This has prompted a renewed focus on strategies to reduce NTSV cesarean delivery rates, specifically emphasizing the use of OVD as a mitigating approach [7, 8, 9, 10, 11, 12, 13]. However, anecdotal concerns from our providers have emerged regarding the preservation of technical proficiency in OVD and the associated risk of increased complications, specifically obstetric anal sphincter injuries (OASIS).

Currently, contemporary data on national trends of OVD and OASIS rates after a movement to reduce NTSV cesarean deliveries in the United States are sparse. Therefore, we conducted a cross‐sectional analysis using national birth data to evaluate temporal trends in OASIS outcomes associated with OVD. This study had three objectives. The first objective was to explore temporal trends of OVD and OASIS among deliveries occurring vaginally. The second objective was to examine the associations between mode of delivery and OASIS as the primary focus, while also exploring the association of other clinical and demographic factors as a secondary focus. The third objective was to estimate temporal trends in the adjusted likelihood of OASIS stratified by type of OVD. We hypothesize that national OVD rates have fallen and, at the same time, the likelihood of OASIS with OVD has increased.

## 2 MATERIALS AND METHODS

This is a cross‐sectional study using 2016– 2023 United States National Vital Statistics System (NVSS) Birth Data. A standardized birth certificate form was introduced in 2003 and by 2016 was implemented universally; therefore, we limited our analysis from 2016 onwards [14, 15, 16]. The data utilized for this study are publicly available by the Centers for Disease Control and Prevention after assembly by the National Center for Health Statistics for research purposes, and prior studies have been performed to ensure the validity of the data being stored [17, 18, 19]. Given the publicly accessible de‐identified data, this study did not require Institutional Review Board oversight.

**FIGURE 1:**
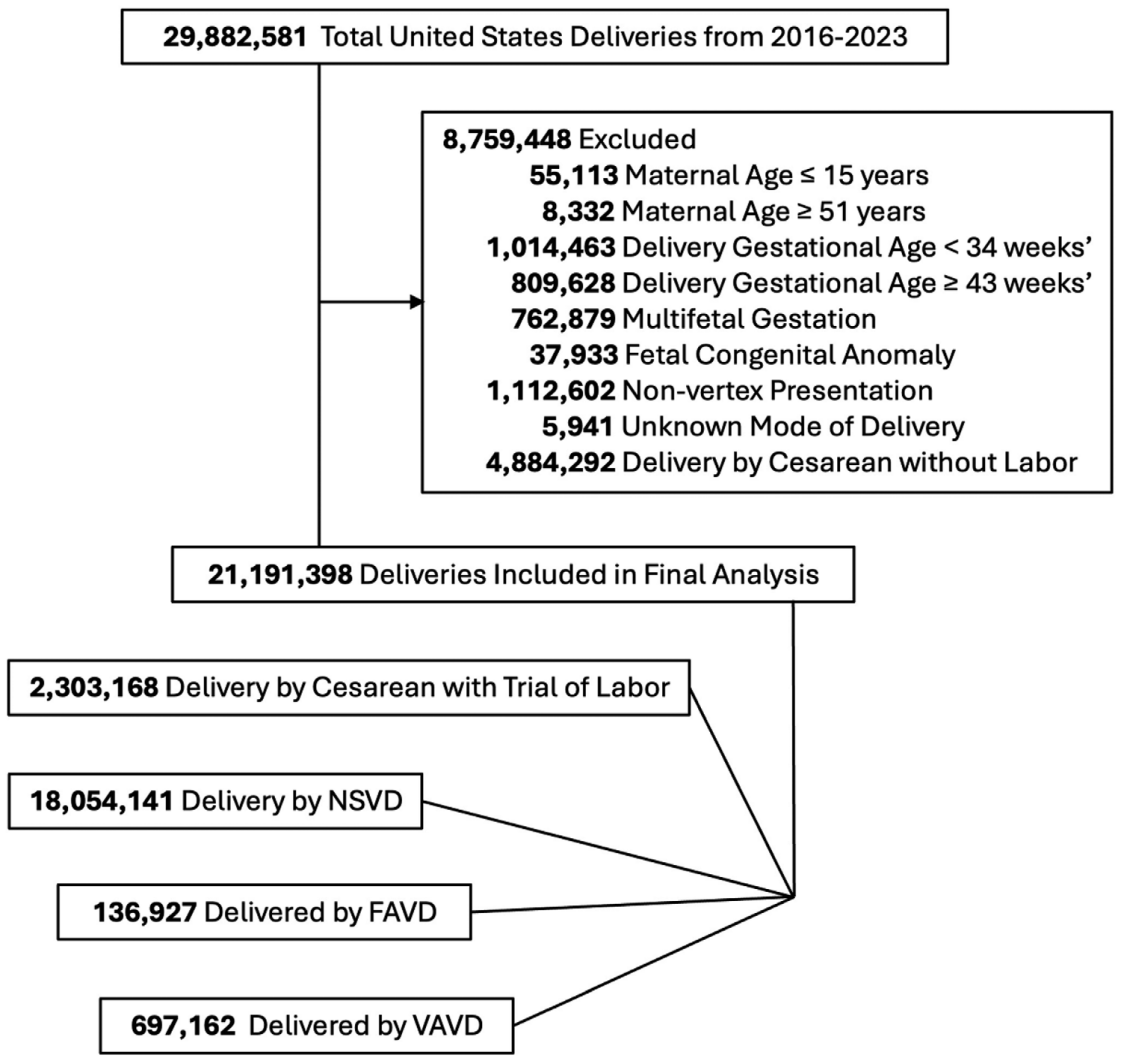
Study flow diagram.

All liveborn deliveries undergoing trial of labor from 34 weeks and 0 days to 42 weeks and 6 days gestational age in the United States between 2016 and 2023 to patients aged 16–50 years were included for this analysis (Figure 1). Our analysis was limited to late preterm and term gestations owing to the relative and absolute contraindications associated with OVD in the preterm period. Deliveries with multifetal gestations, fetal anomalies, non‐vertex presentations, unknown mode of delivery, and those who underwent cesarean delivery without trial of labor were excluded (Figure 1). The primary exposure was mode of delivery, categorized as spontaneous vaginal delivery (SVD), cesarean delivery after trial of labor, FAVD, VAVD, and an OVD composite (a combination of FAVD and VAVD). Our primary outcome metric was the diagnosis of OASIS at time of delivery. In the NVSS birth data, OASIS is defined by the presence of any 3rd or 4th degree laceration and clinical granularity for this definition is unavailable as the outcome is binary.

Baseline demographics were limited to those available on the standardized birth certificate form and included maternal age, body mass index (BMI) at the time of delivery, self‐reported maternal race and ethnicity, health insurance type, parity, gestational age at delivery, and birthweight. Clinical factors included gestational and pregestational diabetes, chronic hypertension, hypertensive disorders of pregnancy (including gestational hypertension and pre‐eclampsia), prior cesarean delivery, and induction or augmentation of labor.

Maternal age was categorized into the following groups: 16–19, 20–24, 25–39, 30–34, 35–39, 40–44, and 45–50. BMI was categorized into standardized groupings as follows: underweight (BMI < 18.5 kg/m^2^), normal (BMI 18.5–24.9 kg/m^2^), overweight (BMI 25–29.9 kg/m^2^), class I obesity (BMI 30–34.9 kg/m^2^), class II obesity (BMI 35–39.9 kg/m^2^), and class III obesity (BMI ≥ 40 kg/m^2^). Health insurance was dichotomized into private insurance and other, which included Medicaid, self‐pay, Indian health service, Tricare, other government, or unknown. Self‐reported maternal race and ethnicity into the following groupings: non‐Hispanic White, non‐Hispanic Black, non‐Hispanic American Indian or Alaskan Native, non‐Hispanic Asian, non‐Hispanic Native Hawaiian or Pacific Islander, non‐Hispanic more than one race, Hispanic, or unknown. Race and ethnicity were included and analyzed not as a biological construct but to evaluate potential disparities in both exposures and outcomes due to racism and structural or social determinants of health [20, 21, 22].

For the first objective estimating temporal trends of OVD and OASIS, we reported the proportion of deliveries by year by mode of delivery. Rates of combined OVD, FAVD, VAVD, and cesarean after trial of labor were calculated and trend analysis was conducted using the National Cancer Institute's Joinpoint Regression Program [23]. The proportion of vaginal deliveries affected by OASIS was similarly calculated and stratified by mode of delivery with accompanying trends analysis. Trends were presented as proportion by year and the average annual percent change (AAPC) with 95% confidence intervals (CI) were determined.

For the second objective examining associations between OASIS and clinical factors, we conducted an analysis of factors stratified by the presence or absence of OASIS. We utilized chi‐squared and ANOVA to compare categorical and continuous variables, respectively. We fit unadjusted and adjusted logistic regression models to primarily assess the relationship between OASIS and mode of delivery, adjusting for maternal age category at delivery, BMI category at delivery, maternal self‐reported race/ethnicity, payer status, parity, pre‐gestational and gestational diabetes, chronic hypertension, pre‐eclampsia spectrum, induction or augmentation of labor, birthweight over 4000 g, and year of delivery. The variables for our adjusted analysis were derived from factors associated with OASIS in the literature and factors associated with potential disparities in maternal care. Unadjusted odds ratios (OR) and adjusted odds ratios (aOR) with 95% CI were calculated as measures of association. As sub‐analyses, we repeated the analysis above stratified by nulliparity or multiparity given these populations have difference baseline incidence of OVD and risk of OASIS.

For the third objective describing the temporal trend in adjusted likelihood of OASIS, we first stratified our population by type of OVD (FAVD or VAVD) and then ran each regression model using year of delivery as an exposure variable and the same covariates in the model as noted in objective two. The aOR and 95% CI were reported for each year to demonstrate the change in OASIS odds.

In all analyses, a *p* value of less than 0.05 or 95% CI not including 1.00 indicated statistical significance. All other statistical analyses were performed using STATA IC Version 15.1.

## 3 RESULTS

Between 2016 and 2023, there were 29,882,581 live births in the United States. After exclusions, 21,191,398 live births were included in our final analysis of which 18,054,141 (85.2%) were SVD, 2,303,168 (10.9%) were cesarean after trial of labor, 136,927 (0.6%) were FAVD, and 697,162 (3.3%) were VAVD (Figure 1). Baseline demographics and characteristics showed statistically significant differences between deliveries complicated with and without OASIS with regard to maternal age, maternal BMI, maternal race/ethnicity, private insurance, nulliparity, pregnancy comorbidities, induction or augmentation of labor, gestational age, and birthweight (Table 1).

**TABLE 1:** Baseline demographics and characteristics. Categorical variables are reported as *n* (%). Continuous variables are reported as mean ± standard deviation.

	No OASIS (*n* = 18,657,102)	OASIS (*n* = 216,815)	*p* value
Mode of delivery			
SVD	17,876,949 (95.8)	163,533 (75.4)	
OVD (composite)	780,153 (4.2)	53,282 (24.6)	
FAVD	120,114 (0.6)	16,665 (7.7)	
VAVD	660,039 (3.5)	36,617 (16.9)	
Maternal age categories			<0.01
Age 16–19	949,344 (5.1)	9,905 (4.6)	
Age 20–24	3,801,606 (20.4)	36,654 (16.9)	
Age 25–29	5,486,431 (29.4)	67,135 (31.0)	
Age 30–34	5,358,980 (28.7)	71,355 (32.9)	
Age 35–39	2,552,478 (13.7)	27,513 (12.7)	
Age 40–44	482,456 (2.6)	4,044 (1.9)	
Age ≥ 45	25,807 (0.1)	209 (0.1)	
BMI categories			<0.01
BMI < 18.5	629,363 (3.4)	8,553 (3.9)	
BMI 18.5–24.9	8,156,270 (43.7)	112,552 (51.9)	
BMI 25.0–29.9	4,928,832 (26.4)	55,288 (25.5)	
BMI 30–34.9	2,631,902 (14.1)	22,663 (10.5)	
BMI 35–39.9	1,189,822 (6.4)	8,791 (4.1)	
BMI ≥ 40	719,241 (3.9)	4,632 (2.1)	
Unknown BMI	401,672 (2.2)	4,336 (2.0)	
Maternal race			<0.01
Non‐Hispanic White	9,717,999 (52.1)	126,034 (58.1)	
Non‐Hispanic Black	2,438,909 (13.1)	15,846 (7.3)	
Non‐Hispanic American Indian or Alaskan Native	141,956 (0.8)	1,217 (0.6)	
Non‐Hispanic Asian	1,139,306 (6.1)	31,360 (14.5)	
Non‐Hispanic Native Hawaiian or Pacific Islander	46,755 (0.3)	463 (0.2)	
Non‐Hispanic More than One Race	432,198 (2.3)	4,068 (1.9)	
Hispanic	4,572,057 (24.5)	35,541 (16.4)	
Unknown	167,922 (0.9)	2,286 (1.1)	
Private insurance	9,219,137 (49.4)	143,771 (66.3)	<0.01
Nulliparity	7,229,716 (38.8)	165,143 (76.2)	<0.01
Pre‐gestational diabetes	111,711 (0.6)	1,595 (0.7)	<0.01
Gestational diabetes	1,159,332 (6.2)	15,716 (7.3)	<0.01
Chronic hypertension	318,723 (1.7)	3,041 (1.4)	<0.01
Gestational hypertension	1,245,287 (6.7)	17,940 (8.3)	<0.01
Pre‐eclampsia spectrum	32,599 (0.2)	427 (0.2)	0. 01
Prior cesarean	570,946 (3.1)	9,860 (4.6)	<0.01
Induction of labor	6,490,661 (34.8)	86,251 (39.8)	<0.01
Augmentation of labor	5,037,504 (27.0)	78,361 (36.1)	<0.01
Gestational age	38.9 ± 1.5	39.2 ± 1.4	<0.01
Birthweight	3317 ± 482	3497 ± 469	<0.01
Birthweight > 4000 g	1,239,304 (6.6)	28,399 (13.1)	<0.01

**FIGURE 2:**
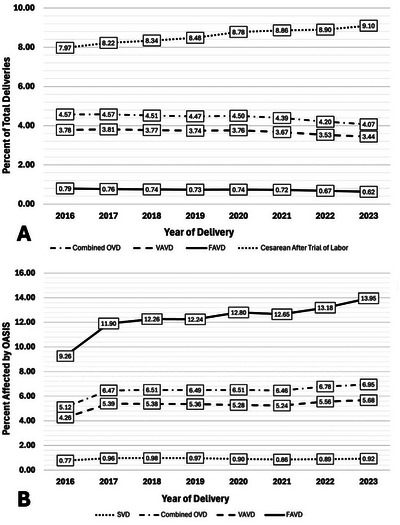
Percent of total deliveries per year for each mode of delivery (A) and percent of patients with each mode of delivery affected by OASIS by year of delivery (B). (A) Significant decreases were appreciated in rate of combined OVD among vaginal deliveries (AAPC: −1.6%, 95% CI: −1.9% to −1.4%), FAVD (AAPC: −3.1%, 95% CI: −3.7% to −2.6%), and VAVD (AAPC: −1.4%, 95% CI: −1.7% to −1.1%). There was no significant change in overall OASIS rates among vaginal deliveries (AAPC: 1.4%, 95% CI: −2.1% to 4.4%). Data for SVD are not included in panel (A). (B) After stratifying, FAVD had significant increased rates of OASIS over time (AAPC: 4.8%, 95% CI: 2.3% to 7.0%) but no significant change seen for OASIS among SVD (AAPC: 1.1%, 95% CI: −2.8% to 4.4%) or VAVD (AAPC: 2.9%, 95% CI: −0.1% to 5.6%).

From 2016 to 2023, the rate of combined OVD among vaginal deliveries significantly decreased from 4.57% to 4.07% (AAPC: −1.6%, 95% CI: −1.9% to −1.4%) (Figure 2). Similarly, FAVD rates significantly declined among live births from 0.79% to 0.52% (AAPC: −3.1%, 95% CI: −3.7% to −2.6%) and VAVD rates significantly declined from 3.78% to 3.44% (AAPC: −1.4%, 95% CI: −1.7% to −1.1%) (Figure 2). During the same period, there was an increase in cesarean delivery after trial of labor from 7.97% to 9.10% (AAPC: 1.8%, 95%CI: 1.5%, 2.2%). There was no significant change in overall OASIS rates among vaginal deliveries (AAPC: 1.4%, 95% CI: −2.1% to 4.4%). However, when stratified by mode of delivery, there was a significant increase in rates of OASIS when delivered by FAVD from 9.26% in 2016 to 13.95% in 2023 (AAPC: 4.8%, 95% CI: 2.3% to 7.0%) (Figure 2). There was no significant change seen for OASIS among SVD (AAPC: 1.1%, 95% CI: −2.8% to 4.4%) or VAVD (AAPC: 2.9%, 95% CI: −0.1% to 5.6%) (Figure 2).

In unadjusted models, mode of delivery (reference SVD) was associated with significantly increased odds of OASIS—FAVD (OR, 15.17; 95% CI, 14.91–15.43) and VAVD (OR, 6.06; 95% CI, 5.99–6.14) (Table 2). Other factors associated with significantly increased odds of OASIS in unadjusted analyses included non‐Hispanic Asian race (reference non‐Hispanic White), private insurance, nulliparity, pre‐gestational diabetes, gestational diabetes, gestational hypertension, pre‐eclampsia spectrum, induction of labor, augmentation of labor, and birthweight over 4000 g at delivery (Table 2). In adjusted models, mode of delivery (reference SVD) retained a significant association with OASIS—FAVD (aOR, 9.50; 95% CI, 9.33–9.66) and VAVD (aOR, 3.90; 95% CI, 3.85–3.94) (Table 2). In this model, significant odds with OASIS were present for non‐Hispanic Asian race and non‐Hispanic Native Hawaiian or Pacific Islander (both reference non‐Hispanic White), private insurance (reference all other payer status), nulliparity, pre‐gestational diabetes, gestational diabetes, gestational hypertension, induction of labor, augmentation of labor, and birthweight over 4000 g (Table 2). With both younger and older age categories and higher BMI classes, there appeared a decreased association with OASIS (Table 2). The sub‐analyses restricted to nulliparous patients only (Table S1) or multiparous patients only (Table S2) yielded similar associations with OASIS by mode of delivery.

**TABLE 2:** Multivariable logistic regression for OASIS among all vaginal births (2016–2023).

	Unadjusted OR (95% CI)	Adjusted OR (95% CI)
Mode of delivery		
SVD	Ref	Ref
VAVD	6.06 (5.99–6.14)	3.90 (3.85–3.94)
FAVD	15.17 (14.91–15.43)	9.50 (9.33–9.66)
Maternal age categories		
Age 16–19	0.85 (0.83–0.87)	0.64 (0.63–0.66)
Age 20–24	0.79 (0.78–0.80)	0.75 (0.74–0.76)
Age 25–29	Ref	Ref
Age 30–34	1.09 (1.08–1.10)	1.10 (1.08–1.11)
Age 35–39	0.88 (0.87–0.89)	1.04 (1.03–1.06)
Age 40–44	0.69 (0.66–0.71)	0.90 (0.87–0.93)
Age ≥ 45	0.66 (0.58–0.76)	0.79 (0.68–0.90)
BMI categories		
BMI < 18.5	0.98 (0.96–1.01)	1.00 (0.98–1.02)
BMI 18.5–24.9	Ref	Ref
BMI 25.0–29.9	0.81 (0.80–0.82)	0.92 (0.91–0.93)
BMI 30–34.9	0.62 (0.62–0.63)	0.77 (0.76–0.78)
BMI 35–39.9	0.54 (0.53–0.55)	0.67 (0.66–0.69)
BMI ≥ 40	0.47 (0.45–0.48)	0.58 (0.57–0.60)
Unknown	0.78 (0.76–0.81)	1.01 (0.98–1.04)
Maternal race/ethnicity		
Non‐Hispanic White	Ref	Ref
Non‐Hispanic Black	0.50 (0.49–0.51)	0.73 (0.71–0.74)
Non‐Hispanic American Indian or Alaskan Native	0.66 (0.62–0.70)	1.02 (0.96–1.08)
Non‐Hispanic Asian	2.12 (2.10–2.15)	1.73 (1.71–1.76)
Non‐Hispanic Native Hawaiian or Pacific Islander	0.76 (0.70–0.84)	1.16 (1.06–1.27)
Non‐Hispanic More than One Race	0.73 (0.70–0.75)	0.83 (0.81–0.86)
Hispanic	0.60 (0.59–0.61)	0.83 (0.82–0.84)
Unknown	1.05 (1.00–1.09)	1.08 (1.03–1.12)
Private insurance^a^	2.01 (2.00–2.03)	1.27 (1.26–1.28)
Nulliparity	5.05 (5.00–5.10)	4.44 (4.39–4.49)
Labor comorbidities		
Pre‐gestational diabetes	1.23 (1.17–1.29)	1.34 (1.28–1.41)
Gestational diabetes	1.18 (1.16–1.20)	1.16 (1.14–1.18)
Chronic hypertension	0.82 (0.79–0.85)	0.97 (0.93–1.01)
Gestational hypertension	1.26 (1.24–1.28)	1.10 (1.08–1.12)
Pre‐eclampsia spectrum	1.13 (1.02–1.24)	1.04 (0.94–1.14)
Induction of labor	1.24 (1.23–1.25)	1.19 (1.18–1.20)
Augmentation of labor	1.53 (1.52–1.54)	1.33 (1.32–1.34)
Birthweight > 4000 g	2.12 (2.09–2.15)	2.71 (2.67–2.74)
Year of delivery		
2016	Ref	Ref
2017	1.26 (1.23–1.28)	1.27 (1.25–1.29)
2018	1.27 (1.25–1.29)	1.28 (1.26–1.31)
2019	1.26 (1.24–1.28)	1.27 (1.25–1.29)
2020	1.19 (1.17–1.16)	1.19 (1.17–1.21)
2021	1.14 (1.12–1.16)	1.14 (1.12–1.16)
2022	1.18 (1.16–1.20)	1.19 (1.17–1.21)
2023	1.20 (1.18–1.22)	1.22 (1.19–1.24)

^a^ Reference for private insurance includes any other payer status including Medicaid, self‐pay, Indian health service, Tricare, other government, other pay, or unknown.

**FIGURE 3:**
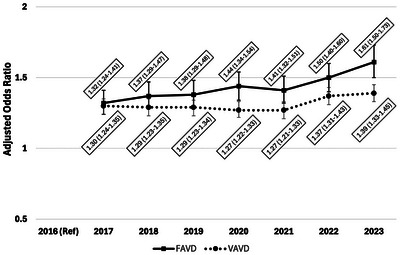
Multivariable logistic regression for OASIS by year stratified by type of OVD using year 2016 as the reference. Adjusted for maternal age, BMI at delivery, maternal race/ethnicity, payer status, nulliparity, pre‐gestational and gestational diabetes, chronic and gestational hypertension, pre‐eclampsia spectrum, induction or augmentation of labor, and birthweight over 4000 g.

When analyzing the adjusted models stratified by type of OVD and using year of delivery as an exposure for our third objective, we observed a temporal increase in odds for OASIS by mode of delivery. For FAVD, we noticed an increase for OASIS from 2016 (aOR, 1.32; 95% Cl, 1.24–1.41) to 2023 (aOR, 1.61; 95% CI, 1.50–1.73) (Figure 3). For VAVD, we noticed a similar but attenuated increase for OASIS from 2016 (aOR, 1.30; 95% CI, 1.24–1.35) to 2023 (aOR, 1.39; 95% CI, 1.33–1.45) (Figure 3).

## 4 DISCUSSION

Our study had three primary findings. First, our national analysis noted a steady decline in OVD rates, primarily driven by declining rates of FAVD. Second, this temporal decline in FAVD rates is accompanied by increases in OASIS among individuals undergoing FAVD. Third, OVD remains a persistent predictor of OASIS among vaginal deliveries and the increased likelihood of OASIS has increased yearly.

Our findings are suggestive of increasing morbidity in the face of declining procedural rates. Consistent with existing literature regarding trends in the United States, our study demonstrated declining rates in OVD, specifically FAVD, and a persistence of OVD as a major risk factor for OASIS [1, 3, 4, 24, 25, 26]. However, the OASIS prevalence was higher in our study than previous time periods [24, 25, 26]. Furthermore, our findings indicate a higher likelihood of OASIS among FAVD in 2023 compared to 2016. This is suggestive of a contemporary temporal increase in the morbidity of the procedure in this subgroup. These two findings point to a worsening in the morbidity of FAVD in the face of declining rates. During the study period, we also note an increase in cesarean after trial of labor, however, these were not isolated to cases of second‐stage arrest.

Other risk factors traditionally associated with OASIS include birthweight, nulliparity, Asian race, induction of labor, augmentation of labor, fetal occiput posterior position, epidural analgesia, and episiotomy [24, 26, 27, 28]. Our findings similarly suggest that birthweight, nulliparity, Asian race, induction of labor, and augmentation of labor are risk factors for OASIS. We are unable to comment on fetal position, epidural analgesia, or episiotomy in our analysis. Our analysis also highlighted a decreased odds of OASIS with younger and older age and higher BMI, which have not been previously reported in the literature [26, 27, 28]. Given that these populations remain at higher risk for cesarean delivery, it is possible the individuals progressing to the second stage and subsequently being offered an OVD are a biased and selected population. Finally, the increased association with OASIS seen with private payer status may highlight disparate care among the population.

As the association with FAVD and OASIS persisted even after inclusion of numerous risk factors in our robust regression model, it is more likely a correlation with FAVD training and comfort than patient risk factors alone. Successful OVD is associated with decreased severe maternal morbidity when compared to cesarean birth and OVD utilization limits the perioperative complications that may arise from cesarean birth [29, 30]. However, severe perineal trauma is associated with immediate sequelae including persistent pain and wound breakdown or infection and long term sequelae including perineal‐rectal or rectovaginal fistulas and urinary or fecal incontinence [31, 32, 33].

One potential explanation of our findings is that decreasing FAVD rates may have led to less familiarity and comfort with FAVD, and a subsequent increase in morbidity. Survey studies have shown that in the United States only 59% of maternal‐fetal medicine fellow physicians and even fewer resident physicians are comfortable with FAVD while program directors overestimate trainee comfort [34, 35, 36, 37, 38]. Given this, the Accreditation Council for Graduate Medication Education (ACGME) has implemented changes to mandated training for graduation without citation. While previously all graduates needed 15 OVD, the new provision allows residents matching into non‐obstetric fellowships such as gynecologic oncology, reproductive endocrinology and infertility, and female pelvic medicine and reconstructive surgery to graduate with fewer OVD. The intent of this change is to allow those more likely to perform OVD in their practice to obtain additional training.

While current efforts by the ACGME in prioritizing OVD for generalist and obstetric fellowship trainees are welcome, future endeavors should also focus on furthering training to improve the clinical proficiency with FAVD and reduce associated OASIS. Alongside this training modification, ACGME mandated OVD and OASIS simulations may be an additional intervention to improve outcomes. In the contemporary period, however, it remains unknown how many practicing obstetricians are comfortable with FAVD and thus, would be able to train residents, fellows, and junior faculty on this delivery modality. Joint collaboration from the ACGME and American College of Obstetricians and Gynecologists (ACOG) should focus on a survey study to determine the proportion of providers who perform FAVD and how many feel comfortable teaching FAVD. Further studies are needed to determine whether a safe re‐vitalization of this skillset is even possible based on the availability of teachers and whether these changes by ACGME will result in improved FAVD skillset in the future.

This study has multiple limitations inherent to the use of birth certificate data. Reliance on birth certificate data is subject to errors in data collection [17, 18, 19]. However, our study largely limited variables included to those acquired direct chart review metrics or binary characteristics with minimal interpretation, thus, limiting user error. While prior studies have not assessed the validity of OVD or OASIS within this dataset, as a significant predictor and an outcome, respectively, with binary measures, they are less subject to misclassification. Owing to unavailable data, we are unable to further characterize many of the granular details of the FAVDs performed that may affect these rates, such as intrapartum characteristics, provider credentials, experience, or type, station and indication for forceps placement, and whether an episiotomy was performed or the type of episiotomy performed (midline or mediolateral). Additionally, further analyses to compare rates of complications between FAVD and cesarean‐related morbidity are unable to be conducted with this data. Such granular analysis may be difficult to perform with currently available data and may be possible in the future with automated data extraction of the electronic medical record. With respect to OASIS, we are unable to separate 3rd and 4th degree lacerations and have no long‐term outcomes. As mentioned earlier, while our data do allow for a robust regression for risk factors for OASIS, we are unable to comment on many others. This study also has multiple strengths including use of a large national representative sample, the ability to perform robust trend analyses and regression modeling including multiple risk factors, and the contemporary nature of the data.

## 5 CONCLUSION

Our findings provide data for risk‐based counseling in the second stage of labor and moreover reveal unique contemporary trends. Considering the ongoing decline in FAVD, if we do not adequately intervene urgently and effectively amongst trainees and junior faculty, OVD and specifically FAVD skillsets may become non‐existent. Providing a subset of learners with adequate training may be more optimal than providing sub‐par training to all learners. Whether we have the teacher volume to recover from this decline, and whether implementation of the ACGME modifications results in improved training, remains to be seen.

## CONFLICT OF INTEREST STATEMENT

The authors declare no conflicts of interest.

## Supporting information



Supporting Information

## Data Availability

The data presented for this study are already publicly available.
