# Supplementary material for: Contemporary trends in operative vaginal delivery and obstetric anal sphincter injuries from 2016 to 2023
Source: Pregnancy (Hoboken). 2025 Jun 30;1(4):e70063. doi: 10.1002/pmf2.70063 (PMC12435513; doi:10.1002/pmf2.70063)
Supplement: Supplementary file 1 [file PMF2-1-e70063-s001.docx]

| **Supplementary Table 1. Multivariable logistic regression for OASIS among nulliparous patients only (2016-2023).** | | |
| --- | --- | --- |
|  | **Unadjusted OR** (95% CI) | **Adjusted OR** (95% CI) |
| **Mode of Delivery** |  |  |
| **SVD** | Ref | Ref |
| **VAVD** | 4.10 (4.05-4.15) | 3.70 (3.65-3.75) |
| **FAVD** | 10.11 (9.93-10.30) | 8.90 (8.73-9.07) |
| **Maternal Age Categories** |  |  |
| **Age 16-19** | 0.46 (0.45-0.47) | 0.60 (0.59-0.61) |
| **Age 20-24** | 0.60 (0.59-0.61) | 0.72 (0.71-0.73) |
| **Age 25-29** | Ref | Ref |
| **Age 30-34** | 1.21 (1.19-1.22) | 1.06 (1.05-1.08) |
| **Age 35-39** | 1.15 (1.13-1.17) | 0.97 (0.96-0.99) |
| **Age 40-44** | 1.00 (0.96-1.05) | 0.83 (0.79-0.87) |
| **Age ≥ 45** | 0.96 (0.80-1.16) | 0.72 (0.59-0.87) |
| **BMI Categories** |  |  |
| **BMI <18.5** | 0.92 (0.90-0.94) | 1.03 (1.00-1.05) |
| **BMI 18.5-24.9** | Ref | Ref |
| **BMI 25.0-29.9** | 0.89 (0.88-0.91) | 0.91 (0.90-0.92) |
| **BMI 30-34.9** | 0.74 (0.72-0.75) | 0.77 (0.76-0.79) |
| **BMI 35-39.9** | 0.65 (0.63-0.66) | 0.68 (0.66-0.70) |
| **BMI ≥ 40** | 0.58 (0.56-0.60) | 0.60 (0.58-0.62) |
| **Unknown** | 0.84 (0.81-0.87) | 0.94 (0.91-0.98) |
| **Maternal Race/Ethnicity** |  |  |
| **Non-Hispanic White** | Ref | Ref |
| **Non-Hispanic Black** | 0.52 (0.51-0.53) | 0.71 (0.70-0.73) |
| **Non-Hispanic American Indian or Alaskan Native** | 0.89 (0.83-0.95) | 1.21 (1.14-1.29) |
| **Non-Hispanic Asian** | 1.88 (1.85-1.91) | 1.64 (1.61-1.66) |
| **Non-Hispanic Native Hawaiian or Pacific Islander** | 0.97 (0.87-1.08) | 1.24 (1.11-1.39) |
| **Non-Hispanic More than One Race** | 0.70 (0.68-0.73) | 0.84 (0.81-0.87) |
| **Hispanic** | 0.62 (0.61-0.63) | 0.81 (0.80-0.82) |
| **Unknown** | 1.06 (1.01-1.11) | 1.09 (1.04-1.14) |
| **Private Insurance*** | 1.69 (1.67-1.71) | 1.19 (1.18-1.20) |
| **Labor Comorbidities** |  |  |
| **Pre-gestational Diabetes** | 1.30 (1.22-1.38) | 1.22 (1.15-1.30) |
| **Gestational Diabetes** | 1.31 (1.29-1.34) | 1.15 (1.12-1.17) |
| **Chronic Hypertension** | 0.93 (0.89-0.97) | 0.99 (0.94-1.03) |
| **Gestational Hypertension** | 1.09 (1.07-1.11) | 1.11 (1.09-1.12) |
| **Pre-eclampsia Spectrum** | 0.95 (0.85-1.06) | 0.98 (0.88-1.09) |
| **Induction of Labor** | 1.23 (1.22-1.24) | 1.24 (1.23-1.25) |
| **Augmentation of Labor** | 1.37 (1.36-1.38) | 1.35 (1.34-1.37) |
| **Birthweight >4000g** | 2.75 (2.71-2.79) | 2.71 (2.67-2.76) |
| **Year of Delivery** |  |  |
| **2016** | Ref | Ref |
| **2017** | 1.27 (1.24-1.30) | 1.27 (1.25-1.30) |
| **2018** | 1.28 (1.26-1.31) | 1.28 (1.26-1.31) |
| **2019** | 1.27 (1.25-1.30) | 1.28 (1.25-1.30) |
| **2020** | 1.23 (1.21-1.26) | 1.23 (1.20-1.25) |
| **2021** | 1.20 (1.17-1.22) | 1.19 (1.17-1.21) |
| **2022** | 1.22 (1.20-1.25) | 1.25 (1.22-1.27) |
| **2023** | 1.23 (1.21-1.26) | 1.26 (1.24-1.29) |

*Reference for private insurance includes any other payer status including Medicaid, self-pay, Indian health service, Tricare, other government, other pay, or unknown.

| **Supplementary Table 2. Multivariable logistic regression for OASIS among multiparous patients only (2016-2023).** | | |
| --- | --- | --- |
|  | **Unadjusted OR** (95% CI) | **Adjusted OR** (95% CI) |
| **Mode of Delivery** |  |  |
| **NSVD** | Ref | Ref |
| **VAVD** | 5.67 (5.51-5.84) | 5.23 (5.08-5.38) |
| **FAVD** | 14.52 (13.91-15.16) | 13.90 (13.31-14.52) |
| **Maternal Age Categories** |  |  |
| **Age 16-19** | 0.55 (0.49-0.62) | 0.70 (0.62-0.79) |
| **Age 20-24** | 0.69 (0.66-0.71) | 0.81 (0.78-0.83) |
| **Age 25-29** | Ref | Ref |
| **Age 30-34** | 1.44 (1.41-1.48) | 1.21 (1.18-1.24) |
| **Age 35-39** | 1.50 (1.47-1.54) | 1.18 (1.15-1.21) |
| **Age 40-44** | 1.27 (1.21-1.33) | 1.02 (0.97-1.07) |
| **Age ≥ 45** | 1.20 (0.98-1.46) | 0.90 (0.73-1.10) |
| **BMI Categories** |  |  |
| **BMI <18.5** | 0.82 (0.78-0.87) | 0.90 (0.86-0.96) |
| **BMI 18.5-24.9** | Ref | Ref |
| **BMI 25.0-29.9** | 0.88 (0.87-0.90) | 0.93 (0.91-0.95) |
| **BMI 30-34.9** | 0.70 (0.68-0.71) | 0.77 (0.75-0.79) |
| **BMI 35-39.9** | 0.58 (0.56-0.61) | 0.66 (0.63-0.68) |
| **BMI ≥ 40** | 0.49 (0.47-0.52) | 0.56 (0.53-0.60) |
| **Unknown** | 1.03 (0.97-1.08) | 1.19 (1.12-1.25) |
| **Maternal Race/Ethnicity** |  |  |
| **Non-Hispanic White** | Ref | Ref |
| **Non-Hispanic Black** | 0.56 (0.54-0.58) | 0.76 (0.73-0.78) |
| **Non-Hispanic American Indian or Alaskan Native** | 0.47 (0.41-0.53) | 0.62 (0.55-0.71) |
| **Non-Hispanic Asian** | 2.27 (2.22-2.34) | 2.08 (2.03-2.14) |
| **Non-Hispanic Native Hawaiian or Pacific Islander** | 0.82 (0.69-0.97) | 1.02 (0.86-1.21) |
| **Non-Hispanic More than One Race** | 0.69 (0.64-0.74) | 0.82 (0.77-0.88) |
| **Hispanic** | 0.67 (0.66-0.69) | 0.87 (0.85-0.89) |
| **Unknown** | 1.02 (0.94-1.12) | 1.05 (0.96-1.15) |
| **Private Insurance*** | 1.89 (1.85-1.92) | 1.48 (1.45-1.51) |
| **Labor Comorbidities** |  |  |
| **Pre-gestational Diabetes** | 1.57 (1.44-1.71) | 1.64 (1.51-1.79) |
| **Gestational Diabetes** | 1.24 (1.20-1.28) | 1.19 (1.16-1.23) |
| **Chronic Hypertension** | 0.78 (0.72-0.83) | 0.92 (0.86-0.99) |
| **Gestational Hypertension** | 0.92 (0.89-0.96) | 1.02 (0.98-1.07) |
| **Pre-eclampsia Spectrum** | 1.15 (0.92-1.42) | 1.29 (1.05-1.59) |
| **Induction of Labor** | 1.03 (1.02-1.05) | 1.04 (1.02-1.06) |
| **Augmentation of Labor** | 1.30 (1.27-1.32) | 1.28 (1.25 (1.30) |
| **Neonatal Birthweight >4000g** | 2.68 (2.62-2.74) | 2.67 (2.61-2.73) |
| **Year of Delivery** |  |  |
| **2016** | Ref | Ref |
| **2017** | 1.24 (1.20-1.29) | 1.26 (1.21-1.30) |
| **2018** | 1.27 (1.23-1.32) | 1.29 (1.24-1.33) |
| **2019** | 1.22 (1.18-1.26) | 1.24 (1.20-1.28) |
| **2020** | 1.07 (1.03-1.10) | 1.08 (1.04-1.12) |
| **2021** | 0.98 (0.94-1.02) | 0.99 (0.96-1.03) |
| **2022** | 1.01 (0.98-1.05) | 1.04 (1.00-1.08) |
| **2023** | 1.04 (1.00-1.08) | 1.08 (1.04-1.12) |

*Reference for private insurance includes any other payer status including Medicaid, self-pay, Indian health service, Tricare, other government, other pay, or unknown.
